# Does Laparoscopic and Endoscopic Cooperative Surgery for Gastric Submucosal Tumors Preserve Residual Gastric Motility? Results of a Retrospective Single-Center Study

**DOI:** 10.1371/journal.pone.0101337

**Published:** 2014-06-26

**Authors:** Yohei Waseda, Hisashi Doyama, Noriyuki Inaki, Hiroyoshi Nakanishi, Naohiro Yoshida, Shigetsugu Tsuji, Kenichi Takemura, Shinya Yamada, Toshihide Okada

**Affiliations:** 1 Department of Gastroenterology, Ishikawa Prefectural Central Hospital, 2-1 Kuratsukihigashi, Kanazawa, Ishikawa, Japan; 2 Department of Gastroenterological Surgery, Ishikawa Prefectural Central Hospital, 2-1 Kuratsukihigashi, Kanazawa, Ishikawa, Japan; 3 Department of General Medicine, Ishikawa Prefectural Central Hospital, 2-1 Kuratsukihigashi, Kanazawa, Ishikawa, Japan; University Hospital Llandough, United Kingdom

## Abstract

**Background:**

Laparoscopic and endoscopic cooperative surgery (LECS) is a minimally invasive surgical technique used to resect gastric submucosal tumors with intraluminal growth. Endoscopic submucosal dissection is used to determine the appropriate resection line from within the stomach lumen as it minimizes the stomach wall resection area and prevents postoperative stomach deformity. Although LECS is intended to preserve gastric function, few reports have evaluated postoperative residual gastric motility. Therefore, we conducted a retrospective analysis of patients who underwent LECS to determine the effects of LECS on residual gastric motility.

**Methods:**

Twenty-two patients underwent endoscopy 3 to 12 months after LECS. Patients were evaluated for endoscopic evidence of gastric motility disorder, namely food residue and occurrence/exacerbation of reflux esophagitis. We considered patients with new onset of gastric symptoms and endoscopic evidence of gastric motility disorder to have clinically relevant gastric motility disorder. We described patient characteristics, tumor location, and surgical findings.

**Results:**

Two of 22 patients developed clinically relevant gastric motility disorder after LECS. In one of these patients, the symptoms were not severe; only one had reduced dietary intake and had lost weight. We identified clinically relevant gastric motility disorder in two patients with gastrointestinal stromal tumors located in the lesser curvature of the stomach. The major axis of these two tumors was 34 mm and 38 mm.

**Conclusions:**

Many patients did not have clinically relevant gastric motility disorder after LECS. Further investigation is required to identify predisposing factors for gastric motility disorder.

## Introduction

Surgeons are increasingly using the minimally invasive surgical technique of laparoscopic local resection of the stomach for gastric submucosal tumors (SMTs) and early gastric cancer [1], [2], [3]. Simple wedge resection is easy to perform in most cases of SMTs with extraluminal growth [4]. However, with the laparoscopic approach, it is often difficult to access posterior wall lesions, and stenosis may result when lesions are adjacent to the esophagogastric junction or pyloric ring. Simple wedge resection of SMTs with intraluminal growth may result in excessive gastric mucosal resection, leading to postoperative gastric deformity. For SMTs with intraluminal growth and early gastric cancer, Ohgami et al developed a technique of laparoscopic wedge resection using a lesion-lifting method [5]. Although their method requires resection of a smaller area than does the simple wedge resection technique, because the resection line is not determined from within the gastric lumen, it is difficult to minimize the resected area. Moreover, decreasing the resected area may result in positive surgical margins. In laparoscopic intragastric surgery, the resection line can be determined from within the stomach lumen; however, it is rather difficult to resect tumors located in the anterior wall of the stomach [6], [7].

Accordingly, Hiki et al developed laparoscopic and endoscopic cooperative surgery (LECS) [8], which uses an endoscopic submucosal dissection (ESD) technique [9]. In LECS, the submucosal layer around the tumor is incised and the entire gastric wall is perforated by intraluminal endoscopy. The surgeon then dissects the tumor along this incision line via laparoscopy. This surgical technique avoids unnecessary resection of the gastric wall and decreases the risk of postoperative gastric deformities, thereby diminishing the risk of affecting gastric function. This technique is also applicable to posterior wall lesions and lesions adjacent to the esophagogastric junction or pyloric ring. Tsujimoto et al reported that in their cases, LECS was not associated with postoperative morbidity or mortality, irrespective of the tumor’s location [10]. Since then, other researchers have confirmed that LECS is a relatively safe and minimally invasive treatment modality [10], [11]. To date, however, few researchers have comprehensively assessed residual gastric motility after surgery. Therefore, the aim of this study was to examine the impact of LECS on residual gastric motility after LECS. To achieve this aim, we conducted a retrospective review of patients who had undergone LECS in our hospital with subsequent endoscopy to evaluate gastric motility 3 to 12 months postoperatively.

## Methods

### Patients

Twenty-seven patients underwent LECS between October 2008 and June 2012 at Ishikawa Prefectural Central Hospital. The preoperative workup included upper gastrointestinal endoscopy with endoscopic ultrasound and computed tomography. Twenty-two patients underwent endoscopy to evaluate residual gastric motility 3 to 12 months (median, 5.5 months; interquartile range, 3–12 months) after the surgery. Five patients were excluded: one who had previously undergone gastrectomy, one who had previously undergone gastric ESD, one who could not be evaluated because of the development of cerebrovascular disease, and two who did not consent to postoperative endoscopy. The remaining 22 patients were followed up for a median period of 32 months (range, 6–48 months). All 22 patients had undergone LECS for gastric SMTs with intraluminal growth.

### Ethics Statement

All patients gave written informed consent for their information to be used for research. This study was approved by the ethics committee of Ishikawa Prefectural Central Hospital.

### Surgical Procedure

Experienced laparoscopic surgeons and experienced endoscopists performed all LECS procedures. The basic procedure starts with induction of general anesthesia and distension of the abdomen by insufflation of carbon dioxide (CO_2_), followed by insertion of the first trocar, containing a camera port, into the umbilical region. Additional ports (range, 0–4 ports) are inserted depending on the patient’s condition; considered factors include obesity, tumor location, abdominal adhesion, and others. The number of ports has been reduced in recent years. The endoscope is then inserted, followed by further CO_2_ insufflation through it. The tumor location is confirmed using the endoscopic and laparoscopic monitors. If any part of the greater or lesser omentum is within the resection area, the omentum is detached to expose the tumor, and blood vessels in the resection area are minimally prepared using an ultrasonic device. The periphery of the tumor is then marked using the endoscope. Using the ESD technique, the mucosa lying directly on the marked periphery is cut and the submucosa undermined circumferentially to create the incision line. The seromuscular layer is perforated and dissected along the incision line by endoscopy; the extent of dissection ranges from one-third to three-quarters of the marked area, depending on the patient’s condition. The seromuscular layer of the remaining area is laparoscopically dissected along the incision line using an ultrasonic device, while confirming the incision line from within the abdominal cavity by laparoscopy. The resected specimen is placed in a plastic bag and removed orally or transumbilically. The stomach wall defect is sutured intracorporeally to minimize gastric deformities [12]. The procedure is terminated following endoscopic confirmation of the absence of bleeding, stenosis, and leakage.

### Evaluation of Residual Gastric Motility

Bar-Natan et al reported that gastric motility after gastric surgery returned by 10 weeks postoperatively [13]. In the present study, evaluation of residual gastric motility was based on the findings of the first endoscopy performed under sedation 3 to 12 months after surgery and on symptoms reported at that time. Patients were considered to have clinically relevant gastric motility disorder if they reported onset of new symptoms, such as heartburn, a heavy feeling in the stomach, epigastric pain, or loss of appetite, and had any endoscopic evidence of food residue or the occurrence/exacerbation of reflux esophagitis. Patients with clinically relevant gastric motility disorder underwent follow-up endoscopy 3 months after the first endoscopy. Reflux esophagitis was graded according to the Los Angeles classification [14]. On endoscopy before LECS, no patients were found to have any food residue in their stomachs, three patients were found to have Los Angeles grade A reflux esophagitis (they were not receiving proton pump inhibitors (PPIs) because they had no symptoms of reflux esophagitis), and insertion of the endoscope into the second portion of the duodenum was easy in all patients. Patients were considered to have marked postoperative gastric deformity if insertion of the endoscope into the second portion of the duodenum after surgery was difficult.

## Results

All 22 patients underwent *en bloc* surgical resection and had negative horizontal and vertical surgical margins. No patients required blood transfusion. No resected areas had any stenosis; the only postoperative complication was gastric motility disorder. None of the 22 patients developed tumor recurrence or metastasis after LECS. All patients received PPIs (lansoprazole 30 mg once daily or omeprazole 20 mg once daily or rabeprazole 10 mg once daily) for 2 weeks immediately after surgery. For patients with symptoms such as heartburn or a heavy feeling in the stomach that persisted for >2 weeks after surgery, the PPIs were continued and gastroprokinetic agents (itopride hydrochloride 50 mg three times daily or mosapride citrate 5 mg three times daily) were added. Three patients were taking PPIs and/or gastroprokinetic agents for their underlying diseases preoperatively; in the remaining 19 patients, the PPIs and gastroprokinetic agents were discontinued upon improvement of symptoms. No patients used continuous nonsteroidal anti-inflammatory drugs or antidepressants that could affect gastric emptying. The first endoscopies, performed 3 to 12 months after surgery, revealed that no patient had marked postoperative gastric deformity. These endoscopies revealed a large amount of food residue in one patient and occurrence of Los Angeles grade B reflux esophagitis in another patient. One patient with a large amount of food residue had type 2 diabetes mellitus that was being treated with insulin therapy. Before LECS, her HbA1c value was 6.6%, and she did not have diabetic gastrointestinal autonomic neuropathy. Endoscopy before LECS revealed no food residue or reflux esophagitis. Her tumor was a low-risk gastrointestinal stromal tumor (GIST) with a major axis of 34 mm in the lesser curvature of the stomach. After LECS, she received lansoprazole 30 mg once daily, but she developed a new and severe heavy feeling in the stomach and loss of appetite. Itopride hydrochloride 50 mg three times daily was added from 2 months postoperatively. Three months postoperatively, her symptoms were still severe, and the first endoscopy revealed a large amount of food residue. Six months postoperatively, her symptoms were slightly improved, and the second endoscopy also revealed food residue. However, although her symptoms were slowly improving, they prevented adequate dietary intake, and her body weight had fallen from 57.6 to 47.4 kg by 12 months postoperatively. She was the only patient who lost more than 5% of their preoperative body weight. Another patient with Los Angeles grade B reflux esophagitis had no underlying disease. Her tumor was a low-risk GIST with a major axis of 38 mm in the lesser curvature of the stomach. After LECS, she received lansoprazole 30 mg once daily for 2 weeks immediately after surgery. The lansoprazole was discontinued because her postoperative abdominal symptoms were showing improvement, but her heartburn continued. Three months postoperatively, the first endoscopy revealed Los Angeles grade B reflux esophagitis. Therefore, she began taking rabeprazole 10 mg once daily. Her symptoms then mostly improved, but continued. The second endoscopy at 6 months postoperatively revealed food residue. Twelve months postoperatively, although she did not lose weight, she was unable to discontinue rabeprazole because of persistence of her symptoms. Four patients had persistent symptoms 2 weeks after surgery and required continuation of the oral medications. However, excluding the two above-described patients, 20 patients showed no symptoms 3 months postoperatively and had no endoscopic evidence of gastric motility disorder. With the exception of three patients taking PPIs and/or gastroprokinetic agents preoperatively, 17 patients discontinued their oral medications. No symptoms occurred after discontinuation of the oral medications. Three patients with Los Angeles grade A reflux esophagitis on preoperative endoscopy had no exacerbation of the reflux esophagitis. Based on these findings, we considered two patients to have clinically relevant gastric dysfunction.

The patient characteristics are shown in Table 1. The data are expressed as the median and range. One patient was undergoing chronic hemodialysis for treatment of chronic renal failure. Five patients with preoperative symptoms were diagnosed by workup of their symptoms. Tumor location, pathological diagnosis, and surgical findings are shown in Table 2. Surgical findings included the major axis of the resected specimen, major axis of the tumor, operating time, intraoperative blood loss, postoperative hospital stay, sentinel lymph node biopsy, direction of stomach wall suturing, and evidence of marked postoperative deformity. Intraoperative bleeding occurred in extremely small amounts and could not be accurately quantified in many patients. In one patient with a GIST, two adjacent lesions were found in the greater curvature of the upper third of the stomach. Because these two lesions were resected as a single specimen, the lesions were handled and examined as one case. Sentinel lymph node biopsy was performed in the patient with a carcinoid tumor. The biopsy revealed no lymph node metastasis in the patient. Two patients with clinically relevant gastric dysfunction had GISTs located in the lesser curvature of the stomach. The major axis of these two tumors was 34 mm and 38 mm. Although these two patients with clinically relevant gastric dysfunction had uneventful postoperative courses, their postoperative hospital stays were longer than those of patients without clinically relevant gastric dysfunction.

**Table 1:** Patient characteristics.

	Patients With Clinically Relevant Gastric Dysfunction	Patients Without Clinically Relevant Gastric Dysfunction
	n = 2	n = 20
Sex (male/female)	0/2	10/10
Age, years (median [range])	64.5 (61–68)	52.5 (31–78)
Body mass index, kg/m^2^ (median [range])	24.9 (24.6–25.2)	22.8 (18.1–33.6)
Underlying disease^a^	1	6
Peptic disease	0	2
Diabetes mellitus	1	3
Ischemic heart disease	0	0
Hypertension	0	5
Hepatic disease	0	0
Renal disease	0	1
Preoperative symptoms (yes/no)	0/2	5/15
Epigastric pain	0	3
Heavy feeling in the stomach	0	1
Nausea	0	1

a Individual patients may have multiple diseases.

**Table 2:** Clinicopathologic characteristics of the tumors and operative data.

	Patients With Clinically Relevant Gastric Dysfunction	Patients Without Clinically Relevant Gastric Dysfunction
	n = 2	n = 20
Tumor location		
Upper third, lesser curvature	1	0
Middle third, lesser curvature	1	2
Upper third, greater curvature	0	3
Middle third, greater curvature	0	2
Lower third, greater curvature	0	2
Upper third, anterior wall	0	2
Middle third, anterior wall	0	4
Middle third, posterior wall	0	5
Pathological diagnosis		
GIST, low-risk	2	8
Carcinoid tumor	0	1
Aberrant pancreas	0	3
Leiomyoma	0	3
Glomus tumor	0	2
Schwannoma	0	2
Granuloma	0	1
Major axis of the resected specimen,mm (median [range])	47.5 (40–55)	40.5 (24–63)
Major axis of the tumor, mm(median [range])	36.0 (34–38)	25.5 (10–39)
Operating time, minutes(median [range])	167.5 (120–215)	137.5 (90–210)
Intraoperative blood loss, ml(median [range])	<5 (1–5)	<5 (1–10)
Postoperative hospital stay, days (median [range])	13.5 (13–14)	8 (8–12)
Sentinel lymph node biopsy	0	1
Direction of stomach wall suturing(long axis/short axis)	1/1	8/12
Marked postoperative deformity	0	0

GIST = Gastrointestinal stromal tumor.

## Discussion

In our medical institution, LECS is performed mainly in patients with SMTs with intraluminal growth. Because LECS opens the stomach wall, resulting in scattering of the gastric contents around the abdominal cavity, this surgical technique is contraindicated in patients with ulceration or tumor exposure in the cupulate part of an SMT because of the possibility of dissemination. According to the Japan Society of Clinical Oncology Clinical Practice Guidelines for GIST, LECS may be indicated for SMTs with diameters of ≤5 cm [15]. In the present series, LECS was performed in 22 patients with SMTs. None of the patients required discontinuation of LECS and conversion to open abdominal surgery. Additionally, there were no postoperative complications other than gastric motility disorder. Therefore, we consider LECS to be a safe and useful treatment method.

Very few studies have evaluated residual gastric motility after local resection of the stomach [3], [16], [17]. Most of the patients in these studies had favorable postoperative gastric motility, but some had reduced dietary intake because of epigastric symptoms. Tsujimoto et al reported that of 20 patients who underwent LECS, none developed malnutrition [10]. Additionally, postoperative endoscopy revealed no evidence of gastric motility disorder, such as food residue or reflux esophagitis [10]. However, that study included only two patients with lesions located in the lesser curvature, and the authors did not provide a detailed description of the postoperative symptoms. Kang et al reported that of 101 patients who underwent LECS, none of the patients with preservation of the cardia and pylorus experienced postoperative epigastric symptoms [18]. However, that study included operative methods such as distal gastrectomy and proximal gastrectomy other than local resection; additionally, postoperative gastric motility was not evaluated by examination techniques such as endoscopy.

In our study, clinically relevant gastric motility disorder occurred in two of the four patients with tumors of the lesser curvature, but did not occur in any of the patients with tumors located in other areas of the stomach. Therefore we consider the possibility that resection of tumors in the lesser curvature may lead to gastric motility disorder. We believe that a potential influence on gastric motility after LECS is resection of Latarjet’s branch of the vagal nerve and gastric deformity. Kubota et al performed local gastric resection of either the lesser or the greater curvature in dogs and physiologically evaluated postoperative residual gastric motility [19]. The authors reported that resection of the lesser curvature involves resection of Latarjet’s branch of the vagal nerve, which is distributed in the lesser curvature, and decreased gastric motor activity, which probably resulted in impaired residual gastric motility. Fukumoto et al reported that shortening of the lesser curvature is the cause of delayed gastric emptying [20]. In our study, postoperative deformity was not evaluated in detail. It is possible that resection of the tumor in the lesser curvature resulted in shortening of the lesser curvature in patients with clinically relevant gastric dysfunction.

The major axis of the tumor in the two patients with clinically relevant gastric dysfunction was relatively long. In the patient with the 38-mm-diameter GIST, the lateral margin of the specimen was 17 mm long, which is rather long. It is possible that tumor resection with a minimum lateral margin may have improved the outcome. In the patient with the 34-mm-diameter GIST, the lateral margin of the specimen was 6 mm long, which is an appropriate length. However, this patient had clinically relevant gastric dysfunction. We believe that tumor size may affect gastric motility after LECS.

Although some studies have shown that PPIs and gastroprokinetic agents are effective against residual postoperative gastric motility, no consensus has been reached [21], [22]. In this study, despite oral medication, the two patients with clinically relevant gastric dysfunction experienced symptoms and had endoscopic confirmation of gastric motility disorder. Because none of the patients without clinically relevant gastric dysfunction reported any symptoms after discontinuation of oral medications, the oral medications were not masking gastric dysfunction. However, the present findings do not determine whether oral medications alleviate or prevent gastric dysfunction, an area that needs further investigation.

This study has several limitations. The assessment of symptoms was based on spontaneous reports by patients, and gastric motility was not evaluated by a further examination technique that can reliably quantify gastric motility, such as physiological function testing or diagnostic imaging. It is possible that mild gastric motility disorder was not detected, introducing bias. However, two patients certainly developed clinically relevant gastric dysfunction after LECS. Other limitations of this study include its retrospective design, the small number of patients treated at a single center, the short-term follow-up, and the lack of comparison with either open or laparoscopic local resection. This study did not have a standardized follow-up evaluation procedure. However, we consider that this study provides new insights with respect to LECS as a minimally invasive surgical technique. Therefore, a prospective multicenter study is needed to evaluate residual gastric motility after LECS.

In conclusion, this study suggests that although many patients do not develop clinically relevant gastric motility disorder after LECS, a few may develop this complication.
